# Antigenic and Genetic Variability of Human Metapneumoviruses

**DOI:** 10.3201/eid1004.030393

**Published:** 2004-04

**Authors:** Bernadette G. van den Hoogen, Sander Herfst, Leo Sprong, Patricia A. Cane, Eduardo Forleo-Neto, Rik L. de Swart, Albert D.M.E. Osterhaus, Ron A.M. Fouchier

**Affiliations:** * Erasmus Medical Center Rotterdam, Rotterdam, the Netherlands; † University of Birmingham Medical School, Birmingham, United Kingdom; ‡ Vigi Virus, São Paulo, Brazil

**Keywords:** Paramyxoviridae, Pneumovirinae, human metapneumovirus, genetic variability, antigenic variability, serotypes, fusion protein, attachment protein

## Abstract

Human metapneumovirus (HMPV) is a member of the subfamily *Pneumovirinae* within the family *Paramyxoviridae*. Other members of this subfamily, respiratory syncytial virus and avian pneumovirus, can be divided into subgroups based on genetic or antigenic differences or both. For HMPV, the existence of different genetic lineages has been described on the basis of variation in a limited set of available sequences. We address the antigenic relationship between genetic lineages in virus neutralization assays. In addition, we analyzed the genetic diversity of HMPV by phylogenetic analysis of sequences obtained for part of the fusion protein (n = 84) and the complete attachment protein open reading frames (n = 35). On the basis of sequence diversity between attachment protein genes and the differences in virus neutralization titers, two HMPV serotypes were defined. Each serotype could be divided into two genetic lineages, but these did not reflect major antigenic differences.

Human metapneumovirus (HMPV) has recently been identified as a causative agent of respiratory tract illnesses in humans worldwide (*1*–*3*) and is a member of the *Pneumovirinae* subfamily within the *Paramyxoviridae* family (4). The *Pneumovirinae* subfamily consists of two genera: the pneumoviruses and the metapneumoviruses. Human respiratory syncytial virus (HRSV), the major viral cause of severe respiratory tract illnesses in children, is the type species of the pneumoviruses (*5*). Avian pneumovirus (APV), the causative agent of respiratory tract illnesses in turkeys and chickens (*6*), was the sole member of the *Metapneumovirus* genus until the discovery of HMPV (*7*).

For most pneumoviruses, different subgroups or subtypes have been identified. For HRSV, two subgroups have been identified on the basis of differences in nucleotide sequences, reactivity patterns with monoclonal antibodies, and in vitro neutralization assays with subgroup-specific antisera (*8*–*11*). Additional genotypes have been identified within subgroups, largely on the basis of the high variability of the attachment protein gene (*12*,*13*). The fusion (F) and the attachment (G) proteins are the main targets for the neutralizing and protective antibody response (*14*–*16*), with F being one of the most conserved proteins and G the most variable (*17*–*20*). For APV, two different subgroups (A and B) have been defined on the basis of nucleotide sequences of the G protein and neutralization tests by using monoclonal antibodies that also recognize the G protein, but these subgroups belonged to one serotype (*21*). APV type C, a possible second serotype, was identified based on the lack of cross-reactivity with antisera specific for groups A and B, and the nucleotide sequences also proved to be substantially different from strains belonging to group A or B (*22*,*23*). In addition, subgroup D may exist, which contains isolates from France that are not neutralized by monoclonal antibodies raised against viruses belonging to either subgroup A, B, or C (*24*).

For HMPV, two major genetic lineages have been identified worldwide on the basis of analysis of a limited set of sequences (*25*–*27*). One feature of HMPV that poses a challenge in developing a future vaccine is that infections may occur in the presence of preexisting immunity. Very young children (<1 year) have been infected by the virus, and reinfections have also been demonstrated (*28*). HMPV might cause repeated infections throughout life, similar to HRSV, which could be either due to incomplete immunity or to genetic heterogeneity of the virus.

To develop vaccines, the extent of genetic and antigenic variability of the different HMPV transmembrane glycoproteins must be understood. We analyzed the genetic diversity of HMPV by phylogenetic analyses of sequences obtained for part of the F (n = 84) and the complete G open reading frames (ORFs) (n = 35). In addition, we addressed the antigenic relationship between the different lineages with virus neutralization assays using lineage-specific antisera raised in ferrets. Virologic studies have used a definition of a homologous-to-heterologous virus neutralization titer ratio of >16 as a definition for serotypes (*29*). On the basis of our results and the described definition, we now define the two major lineages of HMPV as serotype A and B. In accordance with the definition and our results, the sublineages within each serotype are not identified as different serotypes. At least two serotypes of HMPV are present in the human population, a finding that has implications for developing intervention strategies, such as immunization and vaccination.

## Materials and Methods

### Sample Collection, RNA Isolation, RT-PCR Assays, and Sequencing

HMPV-positive nasopharyngeal aspirate samples were obtained from different cohort studies: 61 samples from the Netherlands, 11 samples from Finland, 8 samples from England, 1 from Hong Kong, and 2 from Brazil. Clinical samples had been obtained from 1981 to 2002. Samples were obtained from young children, infants, adults, the elderly, and immunocompromised persons, who had mild to severe respiratory tract illnesses. Epidemiologic and clinical data for most isolates have been described elsewhere (*30*–*32*).

Similar to the influenza nomenclature, sequences are identified by country of origin, identification number, and year of isolation. RNA isolation was performed as described previously (*25*). cDNA was synthesized at 42°C for 60 min with random hexamer primers (Promega, Leiden, the Netherlands) and superscript II RNase H-reverse transcriptase (RT) (Invitrogen, Merelbeke, Belgium). An aliquot of cDNA was used in a polymerase chain reaction (PCR) assay to amplify the full-length G ORF or a fragment of the F ORF. Primers: SH7: 5′- TACAAAACAAGAACATGGGACAAG-3′ and SH-8 5′-GAGATAGACATTAACAGTGGATT-3′ (G ORF), BF100 5′-CAATGCAGGTAT AACACCAGCAATATC-3′, and BF101 5′-GCAACAATTGAACTGATCTTCAGGAAAC-3′ (F ORF). Thermocycling was performed under the following conditions: 94°C for 1 min, 40°C for 2 min, 72°C for 3 min (40 cycles). When necessary, a nested PCR was performed by using 5 μL of PCR product with primers SH7 and SH8 for the G ORF or primers BF103 5′-ACATGCCAACATCTGCAGGACAAA TAAAAC-3′ and BF104 5′-ACATGCTGTTCACCTTCAACTTTGC-3′ for the F ORF. PCR products were sequenced directly on both strands with multiple primers as described previously (*25*). When identical sequences were obtained (suspicious of laboratory contamination) and to confirm sequence uncertainties such as frame shifts, we repeated the RNA isolation, RT-PCR, and subsequent sequencing with the original materials.

### Phylogenetic Analysis

Nucleotide sequences were aligned with the Clustal W program running within the Bioedit software package, version 5.0.9. Maximum likelihood trees were generated with the Seqboot and Dnaml packages of Phylip version 3.6 by using 100 bootstraps and 3 jumbles. The consensus tree was calculated by using the Consense package of Phylip 3.6 and was subsequently used as usertree in Dnaml to recalculate the branch lengths from the nucleotide sequences. Finally, the trees were rerooted at midpoint by using the Retree software of Phylip 3.6. Trees were visualized with the Treeview 1.6.6 program distributed with Bioedit version 5.0.9 (*33*). Sequences are available from GenBank under accession no. AY295930 to AY296012 (F partial) and AY304360 to AY304362 (complete F for NL/17/00, NL/1/99, and NL/1/94, respectively), AF371337 (complete genome NL/1/00), and AY296014 to AY296047 (complete G regions).

### Virus Preparations and Titrations

Viruses were isolated on tertiary monkey kidney (tMK) cells as previously described (*25*). For each genetic lineage a prototype virus isolate was chosen on the basis of its ability to grow to high titers on tMK cells and to reflect the specific genotype for the lineage. Virus titrations were cultured for 7 days, and infected wells were identified by immune fluorescence assays (IFA) with HMPV-specific polyclonal antiserum raised in guinea pigs. Titers were expressed in 50% tissue culture infectious dose (TCID_50_).

### Antisera

Lineage-specific polyclonal HMPV antisera were raised by infecting ferrets with 1 mL of virus-infected tMK supernatants containing approximately 10^4^–10^5^ TCID_50_ virus. All infections were performed in duplo, and the animals with the highest antibody responses are shown. Serum samples were collected at days 0 and 28 postinfection (ferret 1 and 2) or at days 0 and 21 (ferret 3 to 6). Infections were performed as follows: ferrets 1 and 3: HMPV NL/1/00, prototype virus for lineage A1. Ferrets 2 and 5: HMPV NL/1/99, prototype virus for lineage B1. Ferret 4: HMPV NL/17/00, prototype virus for lineage A2 and ferret 6: HMPV UK/5/01 a virus from lineage B2. Ferrets were housed in isolator cages to avoid cross-infections.

HMPV-specific polyclonal antisera were raised in guinea pigs as previously described (*25*). Antisera raised in separate guinea pigs against viruses from the two main genetic lineages (A and B) were mixed 1:1, and this mixture tested positive against all HMPV isolates in IFA.

### Virus Neutralization Assays

Virus neutralization assays of heat-inactivated (30 min 56°C) ferret serum samples were performed as previously described (*25*). Briefly, twofold serial serum dilutions starting at 1:8 were incubated with approximately 30 TCID_50_ virus. Seven days after infection of tMK cells with the antibody and virus mixture, IFA was performed with the guinea pig antiserum. The virus neutralization titer was defined as the reciprocal of the highest serum dilution at which no positive IFA signal was obtained (depicted as means of duplicate measurements). Each experiment included virus titrations of the working solution of the virus, using twofold dilutions, and 10–100 TCID_50_ per well was considered acceptable.

## Results

### Variation in the Fusion Protein Gene

**Figure 1:**
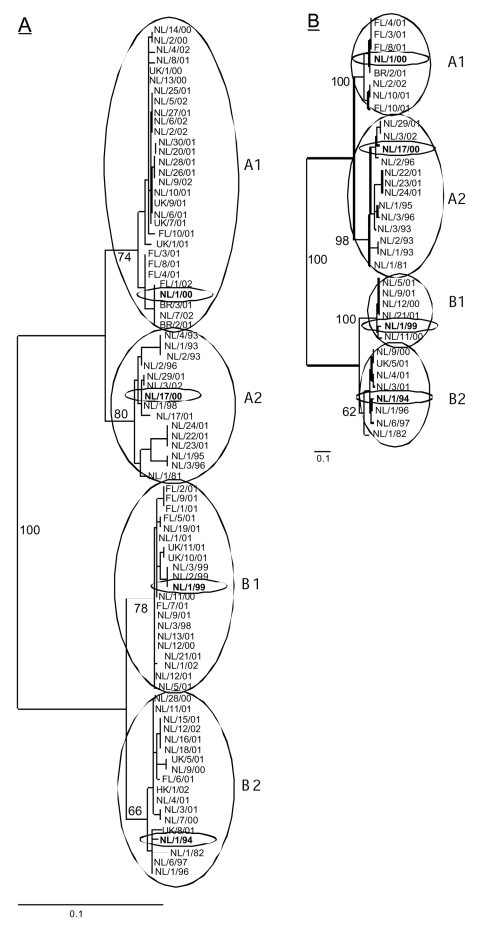
Phylogenetic trees constructed based on the (A) partial F gene (ORF position 780–1,221, n = 84) or (B) the complete G coding region (start G ORF to start L ORF, n = 35). Trees were generated by maximum likelihood analysis using 100 bootstraps and 3 jumbles. The scale representing the percentage of nucleotide changes is shown for each tree. Bootstrap values are based on the consensus trees, and relevant numbers are shown in the tree. The four prototype viruses are shown in boldface, with ovals drawn around them. NL, viruses from the Netherlands; FN, viruses obtained from Finland; UK, viruses obtained from the United Kingdom; HK, viruses obtained from Hong Kong; BR, viruses obtained from Brazil.

Partial F gene sequences (nucleotide [nt] 780–1,221 in the F ORF) were obtained from clinical samples collected from 84 HMPV-infected patients. Phylogenetic analysis of these sequences confirmed two main genetic lineages, A and B. Each of these lineages appeared to consist of two sublineages, which were tentatively named A1, A2, B1, and B2 (Figure 1A).

**Figure 2:**
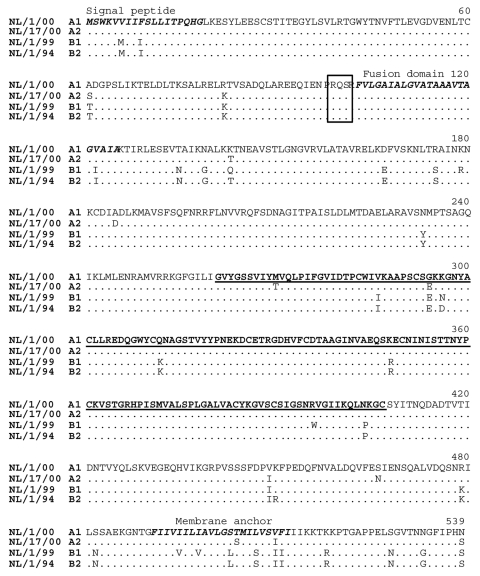
Amino acid sequence comparison of the fusion protein genes of prototype human metapneumovirus isolates of each sublineage. The predicted signal peptide, fusion domain, and membrane anchor are shown in italics in boldface type, the cleavage sites are boxed, and the region sequenced for 84 samples is underlined in boldface type. Periods indicate the position of identical amino acid residues relative to isolate NL/1/00.

Comparison of the sequences showed high percentage identities between members of the same sublineage (nt: 97%–100%, amino acids [aa]: 99%–100%), members of the two different sublineages within each main lineage (nt: 94%–96%, aa: 97%–99%), and between members of the two different main lineages A and B (nt: 84%–86%, aa: 94%–97%). Whereas no specific amino acid residue substitutions could be found between sequences from subgroups A1 and A2, there were 5 specific aa substitutions between sequences from genotypes A and B, and one substitution between B1 and B2 (Table 1). The low variability was also observed when complete F protein genes from prototype viruses for each sublineage were sequenced (Figure 2).

**Table 1:** Lineage-specific amino acid substitutions between the four sublineages in the fusion open reading frame between position 260 and 407

Sublineage	aa_286_	aa_296_	aa_312_	aa_348_	aa_404_
A1	V	K	Q	K	N
A2	V	K	Q	K	N
B1	I	N	K	R	P
B2	I	D	K	R	P

### Variation in the Attachment Protein Gene

**Figure 3:**
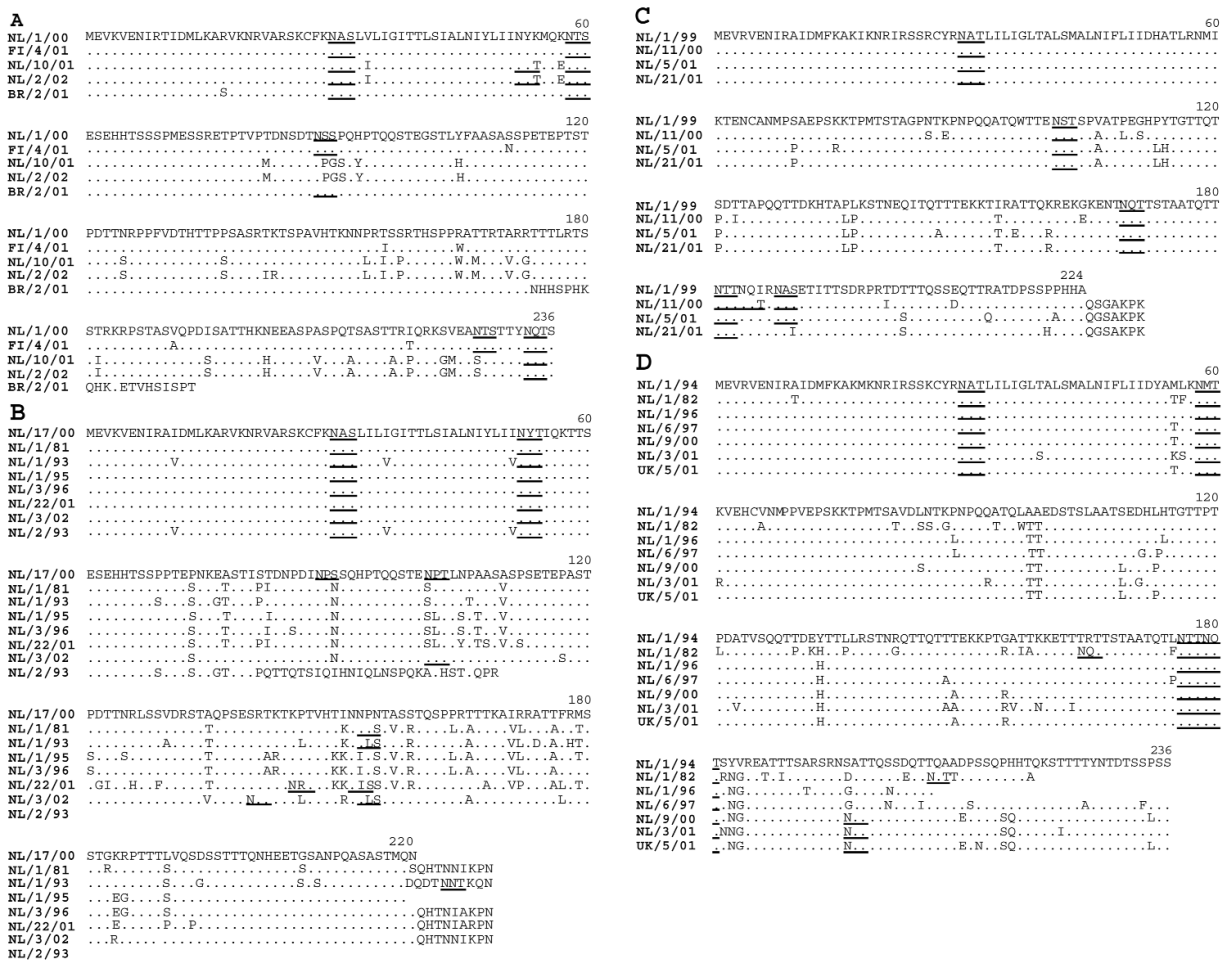
Amino acid sequence comparison of the putative attachment (G) protein of human metapneumovirus strains per genetic sublineage. For each sublineage only representative samples are depicted, resulting in 24 sequences. Representative sequences were chosen, so that from each year in which samples were obtained at least one sequence is depicted, and sequences with only a few amino acid substitutions were omitted. Potential *N-*linked glysosylation sites are underlined, and periods indicate the position of identical amino acid residues relative to the first sequence in each subgroup. Numbers indicate the nucleotide position in the primary G ORF.

Nucleotide sequences of the region between the start codons of the G and the polymerase (L) reading frame were obtained for 35 samples. Phylogenetic analysis showed the same clustering of the sequences over the four sublineages as seen for the F protein gene (Figure 1B). The G region showed some variation in length, from 860 nt to 908 nt. The first 657–708 nt have been described as the putative primary G ORF (4). Alignment of the primary G ORFs showed a variation in length, even for members of the same sublineage, due to single nucleotide substitutions that resulted in premature termination codons (Figure 3). For two samples a change in ORF was observed as a result of an addition (BR/2/01: G at position 519) or a deletion (NL/2/93: C at position 243) of a single nucleotide. These mutations resulted in relatively short G ORFs (NL/2/93: 110 aa; BR/2/01: 193 aa) because of premature termination and in drastic changes in the deduced amino acid sequences of the carboxy-terminus of the G proteins. Comparison of the primary G ORF sequences, excluding sequences of NL/2/93 and BR/2/01 because of the putative frame shifts, showed a relatively high percentage identity between members of the same sublineage (nt: 93%–100%, aa: 75%–99.5%), less identity between members of the two different sublineages within each main lineage (nt: 76%–83%, aa: 60%–75%), and low sequence identity between members of the two different main lineages A and B (nt: 50%–57%, aa: 30%–37%).

The position of the hydrophobic domain, a high percentage of proline, serine, and threonine residues and a cysteine residue at position 27 are features shared by all HMPVs. Whereas the cytoplasmic tail was conserved among all members (58%–70% aa identity), the proposed ectodomains (start aa 51) were quite variable (18%–25% aa identity between lineage A and B). The number and position of potential sites for *N*-linked glycosylation sites varied even within each sublineage, from two to six potential sites, with one located at the proposed cytoplasmic tail conserved among all lineages.

### Geographic and Temporal Distribution

Analysis of HMPV sequences obtained from samples received from different countries indicated that sequences from Finland, the United Kingdom, and the limited sequences from Asia and South America, were found on branches between the Dutch sequences in the F tree, and not as a separate lineage. The variation between sequences obtained from samples from a single country was found in the same range as the variation found between samples obtained from different countries. In agreement with the genetic lineages of HMPV observed worldwide, which usually includes sequences similar to those of isolate NL/1/00 or NL/1/99, geographic clustering does not appear to apply to HMPV.

The different HMPV samples were obtained during the last 20 years with most from 2000 to 2002 and 14 in the 1990s. As indicator for possible fixation of amino acid variation over time, we analyzed the G ORF amino acid sequence of members in sublineage A2 and B1 (containing samples from 1981 to 2002) in more detail. The amino acid sequence variation between the viruses from 1981 and 2001 was in the same range as the variation found between viruses from 2001, and alignments of the sequences did not indicate fixation of amino acid changes between 1981 and 2002. Thus, antigenic drift, as observed for influenza A and B viruses, does not appear to be an important phenomenon for HMPV.

### Antigenic Variation

To address the antigenic variation between the genetic lineages A and B, we raised antisera in ferrets against isolate NL/1/00, the prototype virus for lineage A1, and against isolate NL/1/99, the prototype virus for lineage B1. The serum samples were collected 28 days postinfection and tested in virus neutralization assays against the homologous and heterologous viruses. In three independent experiments, the virus titer used per well varied from 10 to 50 TCID_50_; this variation did not affect the measured virus neutralization titers (Table 2). Ferret 1, infected with the lineage A prototype virus (NL/1/00), showed a 48- to 128-fold higher virus neutralization titer against the homologous virus NL/1/00 than to the heterologous virus NL/1/99. Similarly, ferret 2, infected with the lineage B prototype virus NL/1/99, had a 16- to 96-fold higher homologous than heterologous virus neutralization titer.

**Table 2:** Homologous and heterologous virus neutralizing antibody titers of serum samples obtained from ferrets infected with HMPV viruses belonging to different genetic sublineages^a,b^

Experiment	Virus used in virus neutralization	TCID_50_/well	Ferret 1 NL/1/00 [A1]	Ferret 2 NL/1/99 [B1]
1	NL/1/00	12	**1,024**	32
	NL/1/99	9	16	**512**
2	NL/1/00	30	**1,024**	8
	NL/1/99	20	8	**768**
3	NL/1/00	40	**768**	24
	NL/1/99	25	16	**768**
Ratio A–B			48–128	
Ratio B–A				16–96

^a^Virus neutralizing antibody titers obtained in three independent experiments for serum samples collected 28 days postinfection from ferrets 1 and ferret 2 (infected with NL/1/00 and NL/1/99 respectively). TCID_50_, 50% tissue culture infectious dose; HMPV, human metapneumovirus. ^b^Homologous virus neutralization titers are bold. Values are average of duplicate measurements. Ratios are given as the homologous virus neutralization titers divided by the heterologous virus neutralization titers.

In a second experiment, ferret antisera were raised to viruses from all four sublineages. To measure the most specific serologic response, serum samples were collected 21 days postinfection, after which homologous and heterologous virus neutralization titers were measured (Table 3). Within each main genetic lineage, a high degree of cross-neutralization was observed between viruses from the two sublineages (e.g., A1 vs. A2 and B1 vs. B2), which is reflected in the low ratio between homologous to heterologous virus neutralization titer (0.5 to 3.0). Although serum samples from ferrets 3 to 6 had slightly lower homologous virus neutralization titers than those of ferrets 1 and 2, serum samples raised against viruses from the main lineage A still showed a 12- to 24-fold higher virus neutralization titer against the lineage A viruses than to lineage B viruses. Similarly, serum samples raised against viruses from lineage B had a 16- to 43-fold higher virus neutralization titer against the lineage B viruses than to lineage A viruses.

**Table 3:** Homologous and heterologous virus neutralizing antibody titers of sera obtained from ferrets infected with HMPV viruses belonging to different genetic sublineages^a,b^

Virus used in virus neutralization	Ferret 3 NL/1/00 [A1]	Ferret 4 NL/17/00 [A2]	Ferret 5 NL/1/99 [B1]	Ferret 6 UK/5/01 [B2]
NL/ 1/00	**256**	256	6	32
NL/17/00	512	**768**	12	24
NL/ 1/99	16	32	**256**	384
UK/ 5/01	12	64	256	**512**
Ratio A–B Ratio B–A	16–21	12–24	21–43	16–21

^a^Virus neutralizing antibody titers obtained for sera collected 21 days post infection from ferrets 3 to 6 (infected with NL/1/00, NL/17/00, NL/1/99 and UK/5/01, respectively). HMPV, human metapneumovirus. ^b^Homologous virus neutralization titers are bold. Values are average of duplicate measurements. Ratios are given as the homologous virus neutralization titers divided by the heterologous virus neutralization titers.

## Discussion

In this study, the genetic heterogeneity of HMPV was addressed by analysis of the nucleotide and predicted amino acid sequences of part of the F (n = 84), complete F (n = 4), and the complete G (n = 35) protein genes. Phylogenetic analysis of these sequences showed two main lineages (A and B) with each divided into two sublineages (1 and 2). As was described for HRSV and APV, the F protein was highly conserved, which is in agreement with F proteins of pneumoviruses having structural and functional constraints for amino acid mutations (*34*). On the basis of the high percentage sequence identity for the complete F proteins of the prototype viruses of the four lineages, sequences for the complete F proteins of all 84 samples would probably demonstrate similar low variability. In contrast to the F protein, the nucleotide and predicted amino acid sequences of the complete G coding regions showed high sequence diversity (as low as 30%–37% aa identity). Besides the high amino acid sequence variation, we observed variation in length of the different G proteins. Where most of the length variation was due to nucleotide substitutions, two of the samples showed a change in reading frame due to deletion or addition of single nucleotides. Frame shift mutations and use of alternative reading frames have been described for HRSV (*35*–*37*). As described for isolate NL/1/00, the G coding region of the 35 samples sequenced in the present work indicated long alternative ORFs. However, these secondary ORFs varied in length and position compared to the ones described (*4*). Whether premature stop codons, the incidence of frame shift mutations, and possible use of alternative reading frames influence the antigenic properties of the viruses needs to be examined in more detail.

Phylogenetic analyses showed that the HMPV samples obtained from different years and from different countries were randomly distributed over all four sublineages. For HRSV it has also been reported that very similar viruses were isolated at different times and from geographically distant sites (*36*). Different lineages within HRSV subgroup A and B have been found on the basis of the variation in the G protein. Within each subgroup, progressive accumulation of amino acid changes was noted, suggesting that the G protein of HRSV might be susceptible to immune pressure (*36*). Analysis of the amino acid sequences of the HMPV samples described in this study did not indicate such accumulation over time. However the following observations indicate that the variation of the HMPV G protein might occur as a result of immunogenic pressure in a same manner as was postulated for the RSV G protein: 1) most of the amino acid sequence variation was found in the extracellular domain of the G protein, 2) the variation found at the amino acid sequence level was higher than that at the nucleotide sequence level, 3) the number and position of potential glycosylation sites were not conserved, and 4) deletions, additions and substitutions of single nucleotides resulted in premature stop codons and drastic changes of the carboxy terminal of the protein (*18*,*36*). Until a larger number of more chronologically diverse HMPV samples have been examined, this issue remains inconclusive.

To address the antigenic relationship between members of the different HMPV lineages, we tested ferret sera raised against viruses from the four sublineages in virus neutralization assays. Serologic responses upon infections tend to broaden over time. On the basis of the relatively close genetic relationship between sublineages A1 and A2 or B1 and B2, we decided to collect serum samples at an early time point, to obtain large antigenic differences between the four sublineages. The low homologous virus neutralization titers in serum samples collected 21 days postinfection may explain the lower ratio between homologous and heterologous virus neutralization titers as compared to sera collected 28 days postinfection. The studies with serum samples collected at 21 days postinfection showed that viruses within one main lineage (e.g., A1 and A2 or B1 and B2) were antigenically closely related. The difference in homologous and heterologous virus neutralization titers between members of the two different lineages A and B titers (12- to 128-fold higher homologous titer than heterologous titer) indicate a difference in antigenicity between lineage A and B. Classic virology studies have used a definition of a homologous-to-heterologous virus neutralization titer ratio of >16 for defining serotypes. This same definition notes that if neutralization shows a certain degree of cross-reaction between two viruses in either or both directions (homologous-to-heterologous titer ratio of 8 or 16), distinctiveness of serotype is assumed if substantial differences in sequences are observed (*29*). On the basis of our results, and based on the described definition, we propose defining the two main lineages of HMPV as serotypes A and B. The HMPV samples were obtained from different study populations, from different countries, and from patients with a wide spectrum of clinical signs. So far, we have no indication of an association between infection with either of the serotypes and a specific study group or with severity of disease. More epidemiologic studies are needed to address this issue.

The circulation of two serotypes of HMPV might have implications for the development of vaccines. Studies in cynomolgous macaques showed that reinfection is suppressed by high titers of virus neutralization antibodies against the homologous virus and far less by heterologous virus neutralization antibodies (data not shown). So far, one heterologous reinfection has been reported in humans (*28*). However, children approximately >5 years of age have higher virus neutralization antibody titers than those 1–2 years of age (*25*), which suggests that reinfections may occur frequently, most likely with the viruses from the heterologous serotype. For RSV, the importance of difference in antigenicity between the two subgroups regarding protective immunity and vaccine development is still a subject of discussion. However, in animals and humans, the neutralizing capacity against homologous viruses is higher than that against heterologous viruses, and in animals high homologous virus neutralization titers protect against reinfection. In humans, reinfection often occurs with a strain from the heterologous group, and high homologous virus neutralization antibody titers protect against severe infection (*13*). The two serotypes of HMPV might resemble the two subgroups of HRSV in immunogenic properties, although more extensive epidemiologic and immunologic studies have to prove this. The cross-reactive immunity provided by the F protein may be sufficient to overcome the effects of changes in the G protein. For HRSV the immune response against the F protein is cross-reactive between subgroup A and B, whereas the response against the G protein is subgroup (and sometimes even genotype) specific (*14*,*16*,*38*). The prophylactic use of a virus neutralization monoclonal antibody preparation directed against the HRSV-F protein has been shown to decrease the severity of lower respiratory tract diseases caused by both subgroups of RSV (*39*–*41*). In a similar way, the conserved F protein of HMPV could be a target for the development of monoclonal antibodies for treatment of HMPV-infected persons.

Our data support a technical description of two serotypes of HMPV in experimentally infected ferrets. The existence and relevance of these serotypes in other animal species, including humans, has yet to be determined.

Our results in combination with data published by others (*26*,*27*,*42*) demonstrate that HMPV clusters in two globally distributed serotypes. However, the identification of two serotypes does not exclude the possible existence of more serotypes or sublineages. The described viruses were all identified by using primers against conserved regions in the genome of the four prototype viruses, but in order to allow identification of more diverse HMPV strains, virus isolation of original materials is a standard procedure in our laboratory.
